# Supplementary figures and images for: A comprehensive analysis of chemical and biological pollutants (natural and anthropogenic origin) of soil and dandelion (Taraxacum officinale) samples
Source: PLoS One. 2023 Jan 20;18(1):e0280810. doi: 10.1371/journal.pone.0280810 (PMC9858760; doi:10.1371/journal.pone.0280810)

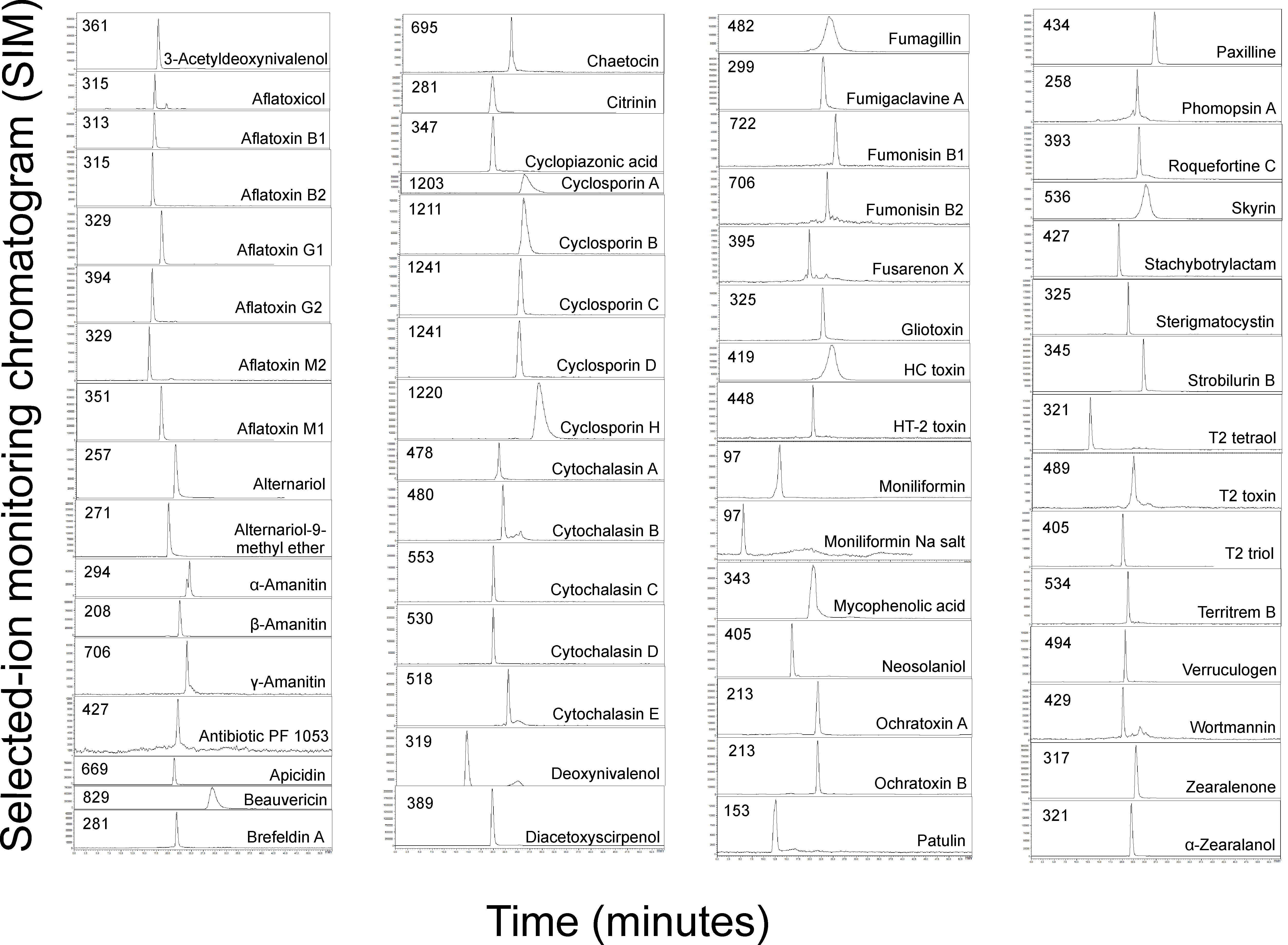

Supplement: S1 Fig — (TIF) [file pone.0280810.s001.tif]
