# Supplementary material for: A comprehensive analysis of chemical and biological pollutants (natural and anthropogenic origin) of soil and dandelion (Taraxacum officinale) samples
Source: PLoS One. 2023 Jan 20;18(1):e0280810. doi: 10.1371/journal.pone.0280810 (PMC9858760; doi:10.1371/journal.pone.0280810)
Supplement: S1 Table — (DOCX) [file pone.0280810.s002.docx]

**Supplementary Table 1. List of standards used, their retention times (RT) and limits of detection (LOD)**

| **No.** | **Standards** | **RT (min)** | **LOD (ng/ml)** |
| --- | --- | --- | --- |
|  | **PAHs:** |  |  |
| 1 | Naphthalene | 14.580 | 0.08 |
| 2 | Naphthalene, 2-methyl- | 18.567 | 0.08 |
| 3 | Naphthalene, 1-methyl- | 19.160 | 0.08 |
| 4 | Acenaphthylene | 23.814 | 0.08 |
| 5 | Acenaphthene | 24.968 | 0.08 |
| 6 | Fluorene | 28.055 | 0.08 |
| 7 | Phenanthrene | 33.767 | 0.08 |
| 8 | Anthracene | 34.038 | 0.08 |
| 9 | Fluoranthene | 41.040 | 0.08 |
| 10 | Pyrene | 42.301 | 0. 2 |
| 11 | Benzo[a]anthracene | 49.796 | 0. 2 |
| 12 | Chrysene | 50.031 | 0. 2 |
| 13 | Benzo[b]fluoranthene | 56.019 | 0. 2 |
| 14 | Benzo[k]fluoranthene | 56.170 | 0.08 |
| 15 | Benzo[a]pyrene | 57.646 | 0.08 |
| 16 | Indeno(1,2,3-cd)pyrene | 63.074 | 0.08 |
| 17 | Dibenz[a,h]anthracene | 63.254 | 0. 2 |
| 18 | Benzo[ghi]perylene | 64.156 | 0. 2 |
|  | **PCBs:** |  |  |
| 19 | 2,2′,5-Trichlorobiphenyl | 13.340 | 0.013 |
| 20 | 2,4′,5-Trichlorobiphenyl | 15.887 | 0.013 |
| 21 | 2,4,4′-Trichlorobiphenyl | 15.906 | 0.007 |
| 22 | 2,2′,5,5′-Tetrachlorobiphenyl | 17.935 | 0.013 |
| 23 | 2,2′,3,5′-Tetrachlorobiphenyl | 19.129 | 0.007 |
| 24 | 2,2′,4,5,5′-Pentachlorobiphenyl | 23.121 | 0.013 |
| 25 | 2,2′,3,4′,5′,6-Hexachlorobiphenyl | 26.765 | 0.007 |
| 26 | 2,3′,4,4′,5-Pentachlorobiphenyl | 26.854 | 0.007 |
| 27 | 2,2′,4,4′,5,5′-Hexachlorobiphenyl | 28.248 | 0.007 |
| 28 | 2,2′,3,4,4′,5′-Hexachlorobiphenyl | 29.873 | 0.013 |
| 29 | 2,2′,3,4,4′,5,5′-Heptachlorobiphenyl | 33.940 | 0.007 |
| 30 | 2,2′,3,3′,4,4′,5,5′-Octachlorobiphenyl | 39.371 | 0.007 |
|  | **Dioxins:** |  |  |
| 31 | 2,3,7,8-TCDF | 28.910 | 0.014 |
| 32 | 2,3,7,8-TCDD | 29.811 | 0.021 |
| 33 | 1,2,3,7,8-Pecdf | 33.647 | 0.016 |
| 34 | 2,3,4,7,8-Pecdf | 34.736 | 0.019 |
| 35 | 1,2,3,7,8-Pecdd | 35.254 | 0.023 |
| 36 | 1,2,3,4,7,8-Hxcdf | 38.816 | 0.025 |
| 37 | 1,2,3,6,7,8-Hxcdf | 38.944 | 0.051 |
| 38 | 1,2,3,7,8,9-Hxcdf | 39.023 | 0.012 |
| 39 | 2,3,4,6,7,8-Hxcdf | 39.830 | 0.019 |
| 40 | 1,2,3,4,7,8-Hxcdd | 40.034 | 0.019 |
| 41 | 1,2,3,6,7,8-Hxcdd | 40.241 | 0.021 |
| 42 | 1,2,3,7,8,9-Hxcdd | 40.663 | 0.021 |
| 43 | 1,2,3,4,6,7,8-Hpcdf | 43.465 | 0.036 |
| 44 | 1,2,3,4,7,8,9-Hpcdf | 43.517 | 0.011 |
| 45 | 1,2,3,4,6,7,8-Hpcdd | 44.961 | 0.022 |
| 46 | 1,2,3,4,6,7,8,9-OCDD | 49.348 | 0.020 |
| 47 | 1,2,3,4,6,7,8,9-OCDF | 49.414 | 0.045 |
|  | **Pesticides:** |  |  |
| 48 | alfa-HCB | 10.880 | 0. 25 |
| 49 | beta-HCB | 12.319 | 0. 25 |
| 50 | Lindane | 12.581 | 0. 25 |
| 51 | delta-BHC | 13.910 | 0. 25 |
| 52 | Heptachlor | 16.513 | 0. 25 |
| 53 | Aldrin | 18.485 | 0. 25 |
| 54 | Heptachlor epoxide | 20.914 | 1.00 |
| 55 | alpha-Endosulfan | 23.042 | 1.00 |
| 56 | Dieldrin | 24.622 | 0. 25 |
| 57 | DDE | 24.758 | 0. 25 |
| 58 | Endrin | 25.900 | 0. 25 |
| 59 | beta-Endosulfan | 26.524 | 1.00 |
| 60 | DDD | 27.288 | 1.00 |
| 61 | Endrin aldehyde | 27.705 | 1.00 |
| 62 | Endosulfan sulfate | 29.156 | 1.00 |
| 63 | DDT | 29.524 | 1.00 |
| 64 | Endrin ketone | 31.867 | 1.00 |
| 65 | Methoxychlor | 33.180 | 1.00 |
