# Supplementary material for: A comprehensive analysis of chemical and biological pollutants (natural and anthropogenic origin) of soil and dandelion (Taraxacum officinale) samples
Source: PLoS One. 2023 Jan 20;18(1):e0280810. doi: 10.1371/journal.pone.0280810 (PMC9858760; doi:10.1371/journal.pone.0280810)
Supplement: S2 Table — (DOCX) [file pone.0280810.s003.docx]

**Supplementary Table 2. Extraction of mycotoxins from *Taraxacum officinale* and soil samples**

| **Sample name** | **Sample mass (g)** | **Extract mass (mg)** |
| --- | --- | --- |
| Flowers Rudenka | 0.5031 | 8.23 |
| Leaves Rudenka | 0.5059 | 3.75 |
| Roots Rudenka | 0.5047 | 5.66 |
| Soil Rudenka | 0.5091 | 4.58 |
| Flowers Warszawa 1 | 0.5012 | 13.88 |
| Leaves Warszawa 1 | 0.5058 | 8.70 |
| Roots Warszawa 1 | 0.5063 | 6.74 |
| Soil Warszawa 1 | 0.5092 | 5.82 |
| Flowers Warszawa 2 | 0.5024 | 7.69 |
| Leaves Warszawa 2 | 0.5077 | 7.48 |
| Roots Warszawa 2 | 0.5020 | 5.64 |
| Soil Warszawa 2 | 0.5034 | 5.71 |
