# Supplementary material for: A comprehensive analysis of chemical and biological pollutants (natural and anthropogenic origin) of soil and dandelion (Taraxacum officinale) samples
Source: PLoS One. 2023 Jan 20;18(1):e0280810. doi: 10.1371/journal.pone.0280810 (PMC9858760; doi:10.1371/journal.pone.0280810)
Supplement: S3 Table — (DOCX) [file pone.0280810.s004.docx]

**Supplementary Table 3. Summary of LC-MS detection conditions of 65 mycotoxins**

| **Mycotoxin** | **CAS / Cat. no.** | **Mycotoxin source** | **Mobile phase** | **Retention Time (min)** | **Ionization mode** | **Fragmentation ions** |
| --- | --- | --- | --- | --- | --- | --- |
| 3-Acetyldeoxynivalenol | 50722-38-8 /  LKT-D1760-M001 | *Fusarium graminearum* | MeOH | 17.714 | positive | 361, 362, 393 |
| Aflatoxicol | 29611-03-8 /  ENZ-CHM104-0001 | *Aspergillus flavus* | ACN | 16.977 | positive | 297, 315, 378, 651 |
| Aflatoxin B1 | 1162-65-8 /  ALX-630-093-M001 | *Aspergillus flavus* | ACN | 16.895 | positive | 313, 330, 346 |
| Aflatoxin B2 | 7220-81-7 /  ALX-630-103-M001 | *Aspergillus flavus* | ACN | 16.373 | positive | 315, 316, 332 |
| Aflatoxin G1 | 1165-39-5 /  ALX-630-104-M001 | *Aspergillus flavus* | ACN | 16.362 | positive | 329, 330 |
| Aflatoxin G2 | 7241-98-7 /  ALX-630-106-M001 | *Aspergillus flavus* | ACN | 15.658 | positive | 331, 376, 394, 395 |
| Aflatoxin M1 | 6795-23-9 /  ALX-630-095-MC01 | *Aspergillus flavus* | MeOH | 18.230 | positive | 329, 351, 352, 374, 383, 397 |
| Aflatoxin M2 | 6885-57-0 /  ALX-630-114-MC01 | *Aspergillus flavus* | MeOH | 17.297 | negative | 329, 330, 361 |
| Alternariol | 641-38-3 /  ALX-350-139-M001 | *Alternaria* sp. | MeOH | 21.447 | negative | 257, 258 |
| Alternariol-9-methyl ether | 23452-05-3 /  LKT-A4678-M001 | *Alternaria* sp. | ACN | 19.974 | negative | 271, 272 |
| α-Amanitin | 23109-05-9 /  ALX-350-270-M001 | *Amanita phalloides* | MeOH | 24.450 | positive | 294, 295 |
| β-Amanitin | 21150-22-1 /  ALX-350-271-M001 | *Amanita phalloides* | MeOH | 22.460 | positive | 191, 208 |
| γ-Amanitin | 21150-23-2 /  ALX-350-272-M001 | *Amanita phalloides* | MeOH | 24.042 | positive | 294, 706 |
| Antibiotic PF 1053 | 147317-15-5 /  ALX-380-147-M001 | *Phoma* sp. | MeOH | 22.083 | negative | 427, 428 |
| Apicidin | 183506-66-3 /  LKT-A6132-M001 | *Fusarium* sp. | ACN | 21.365 | positive | 624, 625, 641, 646, 669, 687 |
| Beauvericin | 26048-05-5 /  BV-B1246-1 | *Beauveria bassiana* | ACN | 29.444 | positive | 784, 785, 801, 802, 806, 829, 830 |
| Brefeldin A | 20350-15-6 /  LKT-B6816-M005 | *Eupenicillium brefeldianum* | MeOH | 21.833 | positive | 281, 282, 298 |
| Chaetocin | 28097-03-2 /  BV-2283-500 | *Chaetomium* sp. | MeOH | 19.092 | negative | 631, 695, 696, 697, 698 |
| Citreoviridin | 25425-12-1 /  LKT-C3576-M001 | *Penicillium citreoviride* | MeOH | 23.383 | negative | 401, 437 |
| Citrinin | 518-75-2 /  BML-CM116-0005 | *Penicillium citrinum* | MeOH | 18.959 | negative | 281, 282 |
| Cyclopiazonic acid | 18172-33-3 /  BML-CA415-0010 | *Penicillium* sp. | MeOH | 19.226 | positive | 304, 347, 353 |
| Cyclosporin A | 59865-13-3 /  BML-A195-0100 | *Fusarium solani* | MeOH | 25.794 | positive | 1163, 1203, 1204, 1205, 1225 |
| Cyclosporin B | 63775-95-1 /  LKT-C9615-M001 | *Trichoderma polysporum* | ACN | 25.740 | positive | 1211, 1212, 1213 |
| Cyclosporin C | 59787-61-0 /  LKT-C9612-M001 | *Fusarium solani* | ACN | 25.117 | positive | 1219, 1237, 1241, 1242, 1243 |
| Cyclosporin D | 63775-96-2 /  BML-T109-0001 | *Fusarium solani* | MeOH | 25.020 | positive | 1234, 1235, 1236, 1239, 1240, 1241 |
| Cyclosporin H | 83602-39-5 /  LKT-C9614-M001 | *Tolypocladium inflatum* | ACN | 29.179 | positive | 1203, 1220, 1221, 1225, 1226 |
| Cytochalasin A | 14110-64-6 /  LKT-C9878-M001 | *Drechslera dematoidea* | ACN | 20.887 | positive | 478, 479, 500, 523, 541, 977 |
| Cytochalasin B | 14930-96-2 /  BML-T108-0005 | *Drechslera dematoidea* | MeOH | 21.688 | positive | 480, 502, 503 |
| Cytochalasin C | 22144-76-9 /  LKT-C9880-M001 | *Metarhizium anisopliae* | ACN | 19.756 | positive | 525, 553, 571, 1037 |
| Cytochalasin D | 22144-77-0 /  LKT-C9881-M001 | *Zygosporium mansonii* | MeOH | 21.851 | positive | 525, 527, 530, 531, 1037 |
| Cytochalasin E | 36011-19-5 /  LKT-C9882-M001 | *Aspergillus clavatus* | MeOH | 22.827 | positive | 474, 518, 519 |
| Deoxynivalenol | 51481-10-8 /  ALX-630-115-M001 | *Fusarium* sp. | MeOH | 14.355 | positive | 319, 320, 335, 337, 351 |
| Diacetoxyscirpenol | 2270-40-8 /  LKT-D3200-M001 | *Fusarium* sp. | MeOH | 19.793 | positive | 386, 389, 390 |
| Fumagillin | 23110-15-8 /  LKT-C9882-M001 | *Aspergillus fumigatus* | ACN | 24.251 | positive | 183, 482, 610 |
| Fumigaclavine A | 6879-59-0 /  ALX-630-110-M001 | *Aspergillus* sp. | MeOH | 22.937 | positive | 299, 300 |
| Fumonisin B1 | 116355-83-0 /  BML-SL220-0001 | *Fusarium moniliforme* | MeOH | 25.646 | positive | 722, 723 |
| Fumonisin B2 | 116355-84-1 /  BML-SL219-0001 | *Aspergillus niger* | MeOH | 23.699 | positive | 704, 706, 711 |
| Fusarenon X | 23255-69-8 /  LKT-F8272-M001 | *Fusarium* sp. | ACN | 22.700 | negative | 331, 395 |
| Gliotoxin | 67-99-2 /  BML-PI129-0002 | *Gladiocladium fimbriatum* | MeOH | 19.724 | negative | 261, 295, 325 |
| HC toxin | 83209-65-8 /  BML-GR320-0001 | *Cochliobolus carbonum* | MeOH | 24.490 | positive | 419, 436, 437, 441 |
| HT-2-toxin | 26934-87-2 /  ALX-630-113-M001 | *Fusarium tricinctum* (*) | ACN | 20.744 | positive | 448, 449, 453 |
| Moniliformin | 31876-38-7 /  LKT-M5853-M001 | *Fusarium* sp. | MeOH | 13.478 | negative | 97 |
| Moniliformin sodium salt | 71376-34-6 /  ALX-630-111-M001 | *Fusarium moniliforme* | MeOH | 5.593 | negative | 59, 97 |
| Mycophenolic acid | 24280-93-1 /  BML-A249-0100 | *Penicillium brevi-compactum* | MeOH | 20.721 | positive | 171, 185, 343 |
| Neosolaniol | 36519-25-2 /  LKT-N1858-M001 | *Fusarium* sp. | MeOH | 16.101 | positive | 405, 406 |
| Ochratoxin A | 303-47-9 /  ALX-630-089-M001 | *Aspergillus ochraceus* | MeOH | 21.679 | positive | 213, 227, 245, 254, 265, 435, 467 |
| Ochratoxin B | 4825-86-9 /  LKT-O0830-M001 | *Aspergillus* sp. | MeOH | 21.690 | positive | 213, 227, 246, 254, 381, 403 |
| Patulin | 149-29-1 /  ALX-270-111-M001 | *Penicillium expansum* | MeOH | 12.608 | negative | 153, 189, 199, 221, 235 |
| Paxilline | 57186-25-1 /  BML-KC155-0005 | *Penicillium paxilli* | MeOH | 26.787 | negative | 434, 494, 869, 929 |
| Penitrem A | 12627-35-9 /  BML-KC157-0001 | *Penicillium palitans* | MeOH | 25.640 | negative | 389, 546, 632, 633, 634 |
| Phomopsin A | 12627-35-9 /  ALX-350-417-M001 | *Phomopsis leptostromiformis* | MeOH | 23.135 | positive | 226, 258, 534 |
| Roquefortine C | 58735-64-1 /  ALX-350-342-MC05 | *Penicillium roqueforti* | MeOH | 23.305 | positive | 390, 393 |
| Skyrin | 602-06-2 /  BV-2043-1 | *Talaromyces* sp. | MeOH | 24.784 | positive | 536, 537, 538 |
| Stachybotrylactam | 163391-76-2 /  ALX-630-112-M005 | *Stachybotrys* sp. | ACN | 18.895 | positive | 386, 427, 428 |
| Sterigmatocystin | 10048-13-2 /  ALX-630-116-M001 | *Aspergillus versicolor* | ACN | 21.059 | positive | 146, 325, 712 |
| Strobilurin B | 65105-52-4 /  ALX-380-144-M001 | *Strobilurus* sp. | MeOH | 24.375 | positive | 345, 347, 377, 379 |
| T2 tetraol | 34114-99-3 /  LKT-T0003-M001 | *Fusarium* sp. | MeOH | 12.733 | positive | 322, 321, 337, 339, 353 |
| T2 toxin | 21259-20-1 /  ALX-630-101-M001 | *Fusarium tricinctum* | MeOH | 22.373 | positive | 389, 484, 489, 490 |
| T2 triol | 34114-98-2 /  LKT-T0004-M001 | *Fusarium* sp. | MeOH | 20.059 | positive | 405, 406, 437 |
| Tenuazonic acid | 610-88-8 /  ALX-350-317-MC05 | *Alternaria* sp. | MeOH | 18.785 | positive | 106, 111, 129, 183, 215, 251 |
| Territrem B | 70407-20-4 /  ALX-630-117-MC05 | *Aspergillus terreus* | ACN | 21.186 | positive | 534, 535, 539, 554, 555 |
| Verruculogen | 12771-72-1 /  LKT-V1870-M001 | *Penicillium verruculosum* | ACN | 20.754 | positive | 428, 445, 491, 494 |
| Wortmannin | 19545-26-7 /  LKT-W5769-M001 | *Penicillium wortmannii* | MeOH | 20.287 | positive | 429, 446, 451, 483 |
| Zearalenone | 17924-92-4 /  ALX-630-105-M010 | *Giberella zeae* | MeOH | 23.099 | negative | 317, 318 |
| α-Zearalanol | 26538-44-3 /  LKT-Z161022-M001 | *Fusarium* sp. | MeOH | 22.322 | negative | 321, 322 |

(*) Semisynthetic, derived from T2 toxin from *Fusarium tricinctum*. ACN –acetonitrile, MeOH – methanol.
