# Supplementary material for: A comprehensive analysis of chemical and biological pollutants (natural and anthropogenic origin) of soil and dandelion (Taraxacum officinale) samples
Source: PLoS One. 2023 Jan 20;18(1):e0280810. doi: 10.1371/journal.pone.0280810 (PMC9858760; doi:10.1371/journal.pone.0280810)
Supplement: S4 Table — (DOCX) [file pone.0280810.s005.docx]

**Supplementary Table 4. List of 18S rDNA sequences used to design PCR reaction primers (1-21) and parasites used as control samples (22-35).**

|  | **Parasite species** | **Adult** | **Eggs** | **Host** | **GenBank accession number** |
| --- | --- | --- | --- | --- | --- |
| 1 | *Ascaridia galli* | - | - | - | EF180058 |
| 2 | *Ascaris lumbricoides* | - | - | - | U94366 |
| 3 | *Ascaris suum* | - | - | - | U94367 |
| 4 | *Dipylidium caninum* | - | - | - | AB731643 |
| 5 | *Enterobius vermicularis* | - | - | - | JF934731 |
| 6 | *Echinococcus granulosus* | - | - | - | AB731639 |
| 7 | *Echinococcus multilocularis* | - | - | - | AB731634 |
| 8 | *Fasciola hepatica* | - | - | - | AJ004969 |
| 9 | *Fascioloides magna* | - | - | - | EF534989 |
| 10 | *Giardia intestinalis* | - | - | - | HQ179639 |
| 11 | *Giardia intestinalis* | - | - | - | AF199444 |
| 12 | *Giardia intestinalis* | - | - | - | HQ179632 |
| 13 | *Giardia intestinalis* | - | - | - | DQ157272 |
| 14 | *Hymenolepis nana* | - | - | - | AY193875 |
| 15 | *Taenia solium* | - | - | - | GQ260091 |
| 16 | *Taenia saginata* | - | - | - | JQ609338 |
| 17 | *Taenia taeniaeformis* | - | - | - | JQ609340 |
| 18 | *Toxocara canis* | - | - | - | U94382 |
| 19 | *Toxocara cati* | - | - | - | EF180059 |
| 20 | *Trichuris trichiura* | - | - | - | DQ118536 |
| 21 | *Trichuris vulpis* | - | - | - | HF586909 |
| 22 | *Ascaridia galli* | + |  | hen |  |
| 23 | *Dipylidium caninum* | + |  | cat |  |
| 24 | *Echinococcus granulosus* | + |  | fox |  |
| 25 | *Echinococcus multilocularis* | + |  | fox |  |
| 26 | *Taenia taeniaeformis* | + |  | dog |  |
| 27 | *Toxocara canis* |  | + | dog | OM964644 |
| 28 | *Toxocara canis* | + |  | dog | OM964643 |
| 29 | *Toxocara cati* |  | + | cat |  |
| 30 | *Toxocara cati* | + |  | cat | OM964645 |
| 31 | *Trichuris* sp. |  | + | cat |  |
| 32 | *Fasciola hepatica* | + |  | wisent |  |
| 33 | *Oxyuridae* sp. |  | + | turtle | OM964642 |
| 34 | *Giardia intestinalis* |  |  | dog | OM964641 |
| 35 | *Giardia intestinalis* |  |  | dried dandelion leaves | OM964640 |
