# Supplementary material for: A comprehensive analysis of chemical and biological pollutants (natural and anthropogenic origin) of soil and dandelion (Taraxacum officinale) samples
Source: PLoS One. 2023 Jan 20;18(1):e0280810. doi: 10.1371/journal.pone.0280810 (PMC9858760; doi:10.1371/journal.pone.0280810)
Supplement: Attachment — Submitted filename: Response to Reviewers.pdf [file pone.0280810.s009.pdf]

Response to Reviewers

Comments to the Author

1. Is the manuscript technically sound, and do the data support the conclusions?

The manuscript must describe a technically sound piece of scientific research with data that supports the conclusions. Experiments must have been conducted rigorously, with appropriate controls, replication, and sample sizes. The conclusions must be drawn appropriately based on the data presented.

Reviewer #1: No

Reviewer #2: Yes

---

2. Has the statistical analysis been performed appropriately and rigorously?

Reviewer #1: Yes

Reviewer #2: Yes

---

3. Have the authors made all data underlying the findings in their manuscript fully available?

The PLOS Data policy requires authors to make all data underlying the findings described in their manuscript fully available without restriction, with rare exception (please refer to the Data Availability Statement in the manuscript PDF file). The data should be provided as part of the manuscript or its supporting information, or deposited to a public repository. For example, in addition to summary statistics, the data points behind means, medians and variance measures should be available. If there are restrictions on publicly sharing data—e.g. participant privacy or use of data from a third party—those must be specified.

Reviewer #1: No

Answer: After addition two supplementary tables (Supplementary Table 6 and 7) containing raw measurements all data underlying the findings presented in the manuscript are fully available.

Reviewer #2: Yes

---

4. Is the manuscript presented in an intelligible fashion and written in standard English?

PLOS ONE does not copyedit accepted manuscripts, so the language in submitted articles must be clear, correct, and unambiguous. Any typographical or grammatical errors should be corrected at revision, so please note any specific errors here.

Reviewer #1: Yes

Reviewer #2: Yes

---

5. Review Comments to the Author

Please use the space provided to explain your answers to the questions above. You may also include additional comments for the author, including concerns about dual publication, research ethics, or publication ethics. (Please upload your review as an attachment if it exceeds 20,000 characters)

Reviewer #1: A comprehensive analysis of chemical and biological pollutants (natural and anthropogenic origin) of soil and dandelion (Taraxacum officinale) samples - pathogenic microorganisms and parasites pose a greater threat
Although the title and purpose of this manuscript is very interesting, I think that in some cases the correct scientific method was not used.
Laboratory methods used for microorganisms are not very clear. Meanwhile, organisms like Francisella grow in specific medium and microaerophilic conditions.

Answer: It is difficult to respond to such a general comment because Reviewer #1 did not mention any specific methodological errors that he believed we had made. In this work, very diverse methods were used, from voltammetric determinations of heavy metals through gas and liquid chromatography coupled with mass spectroscopy (GC-MS and LC-MS), microbiological methods and PCR. All these methods are described in detail in the Materials and Methods section. We expect Reviewer #1 to present specific objections to the methods used by us.

In microbiological studies, we used classical inoculation methods on commercially available media: Columbia Agar plates with 5% sheep blood (CASB) and Sabouraud Glucose Selective Agar plates (SGSA), both from Becton Dickinson. Half of the inoculated CASB plates were kept aerobically, while the second half was kept anaerobically using the anaerobic jar (Roth) and AnaeroGen ™ sachets (Thermo Scientific). All CASB plates were incubated at 37°C for 24 hours. Following this, the number of colony forming units (CFU) was determined using a SCAN 500 automatic bacterial colony counter (Interscience). The bacteria were Gram stained (Gram Stain Kit, Thermo Scientific) and examined under a microscope to distinguish gram-positive and gram-negative colonies and bacterial morphology as this was a prerequisite for strain identification using an automated Vitek-2 system (Biomerieux). Homogeneous bacterial colonies were applied to correctly-selected Vitek-2 disposable cards. The modern, Vitek-2 automated microbiology system is validated and widely used in clinical microbiology to identify various bacterial strains in diagnostic laboratories and hospitals around the world (https://www.biomerieux-usa.com/vitek-2; https://www.ncbi.nlm.nih.gov/pmc/articles/PMC254354/). The composition of the growing media used in the Vitek-2 system cards dedicated various groups of microorganisms is a manufacturer's secret.

Francisella tularensis might be grown in defined media such as Chamberlains or in non-selective media such as Mueller-Hinton broth and a modified Brain Heart Infusion (BHI) broth enhances the growth of this microbe (Morris BJ, Buse HY, Adcock NJ, Rice EW. A novel broth medium for enhanced growth of Francisella tularensis. Lett Appl Microbiol. 2017 Jun;64(6):394-400. doi: 10.1111/lam.12725). We do not know which medium is used in the Biomerieux cards for Francisella tularensis identification (manufacturer secret).

Reviewer #2: The present work is a good effort to detect chemical and biological contaminations in soil and dandelion samples using a wide range of analytical methods (GC-MS, LC-MS, voltammetry, 505 microbiological and microscopic techniques, PCR). I agree with its publication. However, herewith I have few suggestions for its improvement

• Revise the title for clarity

Answer: Revised title: "A comprehensive analysis of chemical and biological pollutants (natural and anthropogenic origin) of soil and dandelion (*Taraxacum officinale*) samples."

• Make abstract and introduction short

Answer: The abstract is only 268 words long and is written in a very concise manner. Attempts to shorten it result in a loss of clarity. The Introduction has been shortened in such a way as not to lose important information and clarity of the message, as well as the justification why the research described in this work was carried out.

• Discussion section needs improvement with more relevant latest articles

Answer: Done

• Avoid use of pronouns (e.g. we, our etc.,) in the text

Answer: Done

• Most of the headings are too lengthy. It would be better to shorten headings

Answer: Where possible, subsection titles have been made shorten.

• Increase visibility of the figures for clarity

Answer: Unfortunately, improving the quality of the Figure 2 and Supplementary Figure 1 is not possible for technical reasons. The quality of the chromatograms depends on the factory software of the GC-MS and LC-MS chromatographs (Shimadzu). The quality of Figures 1, 3, 4 and 5 seems technically satisfactory.

• Supplementary data has captions while captions of main figures are missing

Answer: The titles of the main figures are placed in the main text according to the editorial requirements of PlosOne i.e. Figure 1 lines 130-134, Figure 2 lines 222-224, Figure 3 lines 249-250, Figure 4 lines 301-302, Figure 5 lines 319-329, respectively.
